# Adrenocorticotropic hormone (ACTH) independent Cushing’s syndrome due to unilateral adrenocortical hyperplasia: two case reports

**DOI:** 10.11604/pamj.2021.38.367.26040

**Published:** 2021-04-15

**Authors:** Yousra Aouinati, Amal Mjabber, Nassim Essabah Haraj, Siham El Aziz, Asmaa Chadli

**Affiliations:** 1 Department of Endocrinology, Diabetology and Metabolic Diseases, University Hospital Center Ibn ROCHD, Casablanca, Morocco

**Keywords:** Cortisol, adrenocorticotropic hormone-independent Cushing syndrome, macronodular hyperplasia, case report

## Abstract

Adrenal unilateral macronodular hyperplasia is a rare cause of Cushing's syndrome. We discuss the case of two patients who present Cushing syndrome due to unilateral adrenal hyperplasia. They presented the signs of clinical hypercorticism as well as metabolic, cardiovascular and osteoporotic complications. Both patients presented clinical and laboratory signs of adrenocorticotropic hormone (ACTH)-independent Cushing syndrome with elevated urinary free cortisol (UFC) levels, adrenal computed tomography (CT) scan revealed the appearance of unilateral adrenal adenoma and normal contralateral adrenal gland. Adrenalectomy was performed under laparoscopic surgery; the resected mass was pathologically diagnosed as unilateral nodular adrenal hyperplasia. Unilateral adrenal hyperplasia is a very rare etiology of ACTH-independent Cushing syndrome, often mistaken for adenoma on CT and only pathological examination can confirm the diagnosis.

## Introduction

Cushing's syndrome describes a chronic supraphysiological secretion of cortisol. A primary adrenal etiology is involved in approximately 15 to 20% of Cushing's syndromes. Cortisol is secreted by unilateral adenoma or carcinoma or, more rarely, by micronodular or macronodular hyperplasia [1,2]. Macronodular hyperplasia is an exceptional cause of Cushing Syndrome; we report two cases which were mistaken for adrenal adenomas on CT scan until anatomopathological examination confirmed the diagnosis.

## Patient and observation

**Clinical case 1:** a 60-year-old female patient, who had, during the past year, experienced a weight gain of 15 kilograms, predominantly facial and thoraco-abdominal, with polyuria-polydipsia syndrome and pain in the right hip. She was admitted to our service for diagnosis and treatment. Physical examination on admission showed: a height of 150 centimeters; a weight of 72 kilograms; a waistline of 120cm; a blood pressure of 170/90 mmHg; capillary blood sugar at 2.19 g/l, with a typical clinical picture of hypercorticism with the presence of a painful hip during motion without limitation of movement. The hormonal balance found a urinary free cortisol (UFC) level raised to 4 times the normal, plasma adrenocorticotropic hormone (ACTH) was at the lower limit of the normal value at 14 µg/ml (10-60).

**Figure 1:**
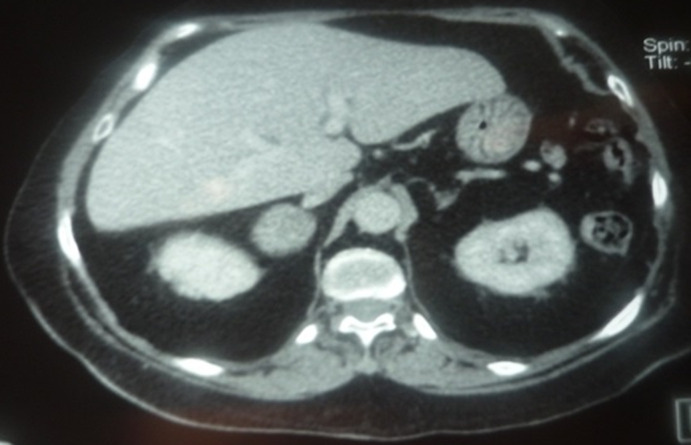
adrenal CT scan, tumor mass at the right adrenal, measuring 46 x 36mm, the left adrenal was not hyperplastic

**Figure 2:**
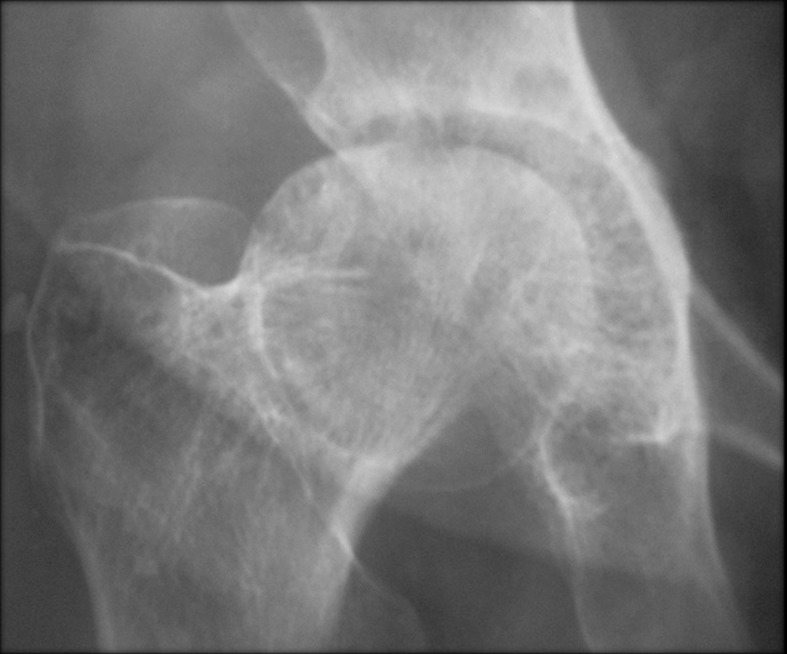
aseptic necrosis of the hip on radiographs

The adrenal CT scan revealed a well-defined right adrenal mass measuring 46 x 33mm, spontaneously hypodense taking the contrast product heterogeneously, the left adrenal was normal non-hypertrophic (Figure 1). As complications of her hypercorticism, the patient had diabetes, dyslipidemia, hyperuricemia, high blood pressure, diffuse bone demineralization complicated by aseptic necrosis of the right femoral head diagnosed on standard radiography of the hip (Figure 2) and pelvic scan and for which she was put on discharge and bisphosphonate treatment for osteoporosis. Once the diagnosis of ACTH independent Cushing's syndrome was reached, a right adrenalectomy was performed by laparoscopy, the postoperative consequences were simple with, as a sign of remission, the occurrence of acute adrenal insufficiency on the third postoperative day, which has been substituted.

Histological examination with complementary immunohistochemical study showed a right adrenal gland measuring 50 x 40 x 25mm, the nodules measure between 0.5 and 1.5cm, with a nodular chamois yellow appearance. Microscopic examination revealed a hyperplasia of the adrenal parenchyma with an increase in the number of spongiocytes, the complementary immunohistochemical study showed an expression of cytokeratin and chromogranin A in favor of hyperplasia. In conclusion, it was macronodular adrenal hyperplasia. Remission was clinically defined by the disappearance of clinical signs of hypercorticism; the patient´s weight decreased from 72 to 56 kilograms; her hyperglycemia showed clear improvement, fasting blood sugar was at 1.10 g/l and the postprandial blood sugar at 1.30 g/l. On the other hand, a residual arterial hypertension,although less severe, remained, measured at 150/80 mmHg. On the paraclinical side, glycosylated hemoglobin was at 7%, while plasma and urine calcium/phosphorus levels, as well as the lipid profile, were normal. Eight A.M. cortisol level was at 38.22 μg/l (62-194) and substitution by Hydrocortisone at a dose of 20 mg/day was maintained.

**Clinical case 2:** a 48-year-old female patient who, since 2009, has been showing weight gain, predominantly on the face and thoraco-abdominal area, with facial erythrosis and joint pain. She was admitted to our department for diagnosis and treatment. Physical examination on admission showed: a height of 145 centimeters; a weight of 82 kilograms; a blood pressure of 180/85 mmHg; capillary blood glucose at 0.91 g/l, with a typical clinical picture of hypercorticism with the presence of pain on palpation of the thoracolumbar spine. A hormonal panel found a UFC level elevated to twice the normal at 520 μg/24h, plasma ACTH was at 18 μg/ml (10-60). Adrenal CT scan showed a right adrenal gland containing a nodule which is enhanced shortly after injection of contrast agent and measures approximately 28 mm, the left adrenal was of normal non-hypertrophic morphology. High blood pressure, left ventricular hypertrophy shown on echocardiography and depression were all complications of hypercorticism that were found in the patient.

Once the diagnosis of Cushing's syndrome was made, a laparoscopic right adrenalectomy was performed. Histological examination showed a right adrenal gland measuring 45 x 30mm. The multiple samples examined show an adrenal parenchyma surrounded by a thin and sometimes moderately thick fibrous shell with emanations towards the parenchyma. On microscopic examination, it was a hyperplasia of the adrenal parenchyma involving the cells of the medulla and the cortex with a predominance of spongiocyte, as well as the presence of small foci of compact cells with nuclear atypia. In conclusion, it was macro nodular adrenal hyperplasia. Five years after the surgery, clinical evolution was marked by the regression of the signs of Cushing syndrome and the persistence of a slight facial erythrosis, the weight went from 82 to 64 kilograms, although the patient kept residual high blood pressure, it was less severe, measured at 160/90 mmHg. On the paraclinical level, plasma and urinary phosphocalcic panel, as well as the lipid panel were normal, UFC was normal at 197 mmol/24h (40-200) and 8 AM cortisol level was also normal at 132.70 ng/l.

## Discussion

ACTH-independent Cushing syndrome by macronodular adrenal hyperplasia was first described in 1964, several series of cases concerning bilateral adrenal hyperplasia (macro or micronodular) were published, moreover unilateral adrenal nodular hyperplasia is a rarer or even exceptional cause of ACTH-independent Cushing syndrome, few studies have been published about this point [1-6]. From an epidemiological point of view, the ACTH-independent Cushing syndrome due to unilateral macronodular adrenal hyperplasia presents itself at extreme ages and mainly between the age of 50 and 60 years [7], our two patients were aged 60 and 48 years old. These are most often sporadic cases; no similar cases in family history have been reported so far. Clinically, the majority of patients present with the typical signs of Cushing syndrome at the time of diagnosis. Biologically, ACTH is generally very low; in our first patient, the ACTH level was at the lower limit whereas it was normal in the second patient, this was also found in a few cases in the literature [8-10].

On CT scans, unilateral adrenal hyperplasia presents as a mass of tissue density, and is often mistaken for adrenal adenoma [11-13]. Adrenalectomy is usually performed by laparoscopy [14,15]. The diagnosis is confirmed postoperatively by the pathological examination [16-18], which shows nodules in an adrenal tissue of yellow-buff color on the macroscopic level as well as the presence of compact and spongiform cells on the microscopic level [19], which is in favor of macronodular hyperplasia the latter must not be confused with bilateral macronodular hyperplasia. However, careful hormonal and radiological monitoring of the remaining adrenal gland should be initiated and maintained over a long period after surgery [20].

## Conclusion

Unilateral adrenal hyperplasia is a very rare etiology of ACTH-independent Cushing syndrome, often mistaken for an adenoma on CT, only pathological examination can confirm the diagnosis.
